# Computed tomography-guided preoperative charcoal tattooing in patients with recurrent prostate cancer after prostatectomy and undergoing pelvic salvage lymphadenectomy

**DOI:** 10.1590/0100-3984.2024.0038

**Published:** 2024-11-18

**Authors:** Juan Bautista Del Valle, Sebastian Gustavo Tirapegui, Juan Cruz Liyo, Matías Adrián Borensztein

**Affiliations:** 1 Hospital Italiano de Buenos Aires, Buenos Aires, Argentina

## INTRODUCTION

Prostate cancer is the most common invasive cancer in men. Although radical prostatectomy is the primary treatment, biochemical recurrence rates can reach 40%^(1)^. In cases of recurrence, local lymph nodes may be involved. Salvage lymphadenectomy has been proposed in patients with biochemical recurrence^(2)^. However, dissecting such nodes can often be a challenging task due to their location or small size. Percutaneous tattooing has been incorporated into surgery to achieve a more accurate localization of affected tissue^(3)^. However, there is limited data in the literature addressing preoperative tattooing in the pelvic region^(4,5)^.

We present the cases of two patients (71 and 60 years of age, respectively) with a history of radically treated prostatic adenocarcinoma who underwent salvage lymphadenectomy. The study was approved by the local institutional review board (Reference no. 6812), and the need for informed consent was waived. Cases were selected in a multidisciplinary meeting. The inclusion criteria were having lymph nodes ≤ 15 mm that were tracer-avid on ^18^F-choline positron-emission tomography/computed tomography (PET/CT) in lymphatic pathways for metastatic prostate cancer (confined to the pelvis) and having been diagnosed with biochemical relapse (prostate-specific antigen level > 0.4 ng/mL). Tattooing was performed by an interventional radiologist with over five years of experience.

## PROCEDURE

Tattooing was performed immediately before surgery. None of the patients had a coagulation disorder or were taking antiplatelet/anticoagulant medications.

**Figure 1:**
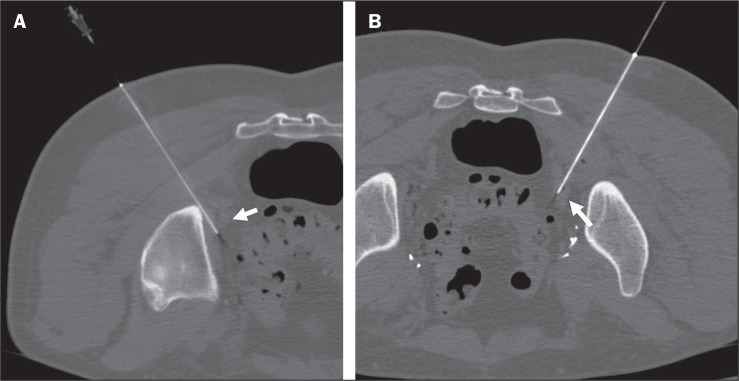
A 71-year-old male patient with a previous prostate tumor (Gleason score of 7 [4+3] and pT3b-pN1-pMx staging), a prostate-specific antigen level of 0.74 ng/mL, one 9-mm right-sided internal iliac lymph node, and one 10-mm left-sided internal iliac lymph node (standardized uptake values of 5.4 and 3.5, respectively). Tattooing was performed by using direct approach with a 21-gauge lumbar puncture needle (arrows in A and B). The final diagnoses were prostatic adenocarcinoma metastasis and a reactive lymph node, respectively.

**Figure 2:**
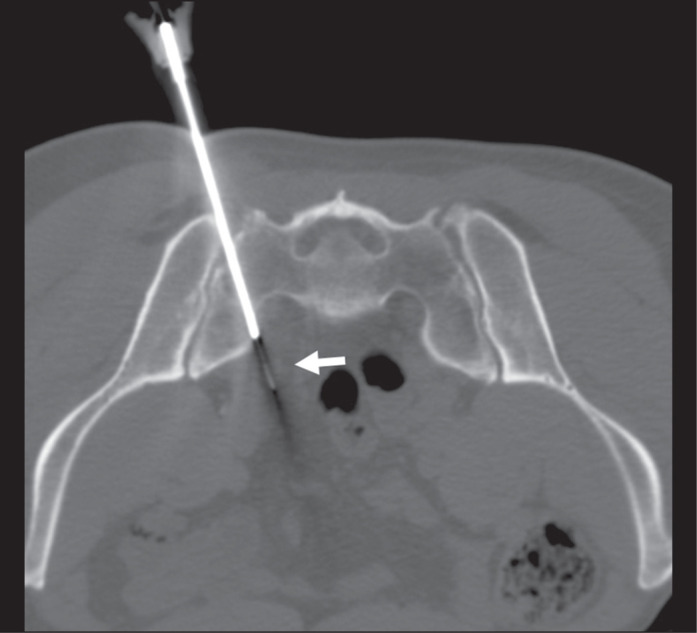
A 60-year-old male patient with a previous prostate tumor (Gleason score of 9 [5+4] and pT3a staging), a prostate-specific antigen level of 0.89 ng/mL, and one 10-mm presacral lymph node (standardized uptake value not available). Tattooing was performed by using a transsacral approach (arrow). The final diagnosis was prostatic adenocarcinoma metastasis.

**Figure 3:**
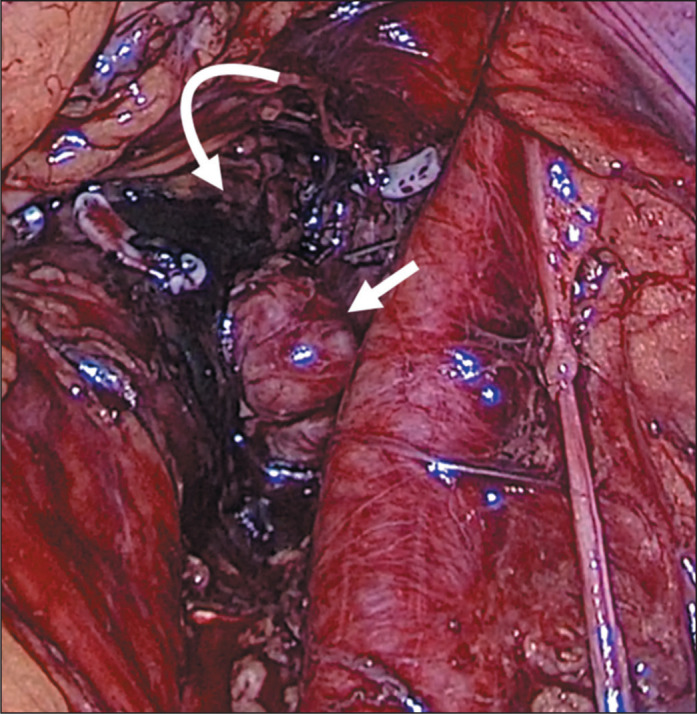
Intraoperative photograph showing the target lymph node (straight arrow) with activated charcoal in its bed (curved arrow).

The tattooing was done in a 16-slice CT scanner with the patient in the prone position. The shortest path to the target was planned, avoiding blood vessels, nerves, and hollow organs. Aseptic technique and 2% lidocaine were used. For direct approaches, 21-gauge lumbar puncture needles were used (Figure 1). If a bone blocked the needle’s trajectory, a transosseous approach was used, with an 11-gauge Jamshidi needle and a Chiba needle (Figure 2). Only the superficial periosteum was anesthetized. Needles were advanced to the surface of the target lymph node, where 4% activated charcoal (Mamograf; Temis Lostalo, Buenos Aires, Argentina) was deposited (Figure 3). Afterward, patients were taken to the operating room.

The time from prostatectomy to salvage lymphadenectomy ranged from 1 to 3 years. All lymph nodes that were tracer-avid on PET/CT were tattooed, identified, and removed during surgery. In one case, two lymph nodes (both tracer-avid on PET/CT) were tattooed via a direct approach and five lymph nodes were dissected; among those five lymph nodes, one was reactive and two were metastatic (one without tracer uptake). In the other case, one tracer-avid lymph node was tattooed via a transosseous approach and four lymph nodes (all metastatic) were dissected.

## COMMENTS

We hypothesize that tattooing the lymph node bed is a simpler surgical identifier than tattooing inside the node. Successful tattooing requires visibility in the surgical field, highlighting the need for meticulous pre-planning and coordination with the surgical team^(3,4)^. Permanent skin tattooing is the primary adverse effect of charcoal-based methods, whereas complications like organ perforation and pain vary depending on the puncture technique and operator experience^(6,7)^.

Although transosseous access might seem to be a painful procedure, it was painless and was consistent with observations from complex biopsies. The technique has diverse applications and is adaptable, with reported variations^(5,8)^.

## CONCLUSION

Lymph node tattooing guided by CT is feasible for salvage lymphadenectomy in patients with small, deep metastatic nodes. More data are needed in order to assess its impact on surgical precision.
